# Balloon Fracturing Valve-in-Valve: How to Do It and a Case Report of TAVR in a Rapid Deployment Prosthesis

**DOI:** 10.1155/2022/4368887

**Published:** 2022-05-04

**Authors:** Rodrigo Petersen Saadi, Ana Paula Tagliari, Carisi Anne Polanczyck, João Carlos Ferreira Leal, Eduardo Keller Saadi

**Affiliations:** ^1^Post Graduate Program in Cardiology and Cardiovascular Science, Federal University of Rio Grande do Sul., Porto Alegre, Brazil; ^2^Post Graduate Program in Cardiology and Cardiovascular Science, Federal University of Rio Grande do Sul. Cardiovascular Surgeon at Hospital São Lucas da PUC-RS, Porto Alegre, Brazil; ^3^Graduate Program in Cardiology, Federal University of Rio Grande do Sul Hospital de Clínicas de Porto Alegre, Porto Alegre, Brazil; ^4^Brazilian Society of Cardiovascular Surgery, São Paulo, Brazil; ^5^Cariovascular Surgery, Federal University of Rio Grande do Sul, Porto Alegre, Brazil

## Abstract

Transcatheter aortic valve replacement (TAVR) to treat degeneration of bioprosthetic heart valves (BHVs), called as valve-in-valve (ViV), is becoming a key feature since the number of BHVs requiring intervention is increasing and many patients are at high risk for a redo cardiac surgery. However, a TAVR inside a small previous cardiac valve may lead to prosthesis-patient mismatch (PPM) and not be as effective as we hoped for. An effective option to decrease the chance of PPM is to fracture the previous heart valve implanted using a high-pressure balloon. By performing a valve fracture, the inner valve ring of small BHVs can be opened up by a single fracture line, allowing subsequent implantation of a properly sized transcatheter heart valve, without increasing substantially the procedure risk. In this article, we provide a step-by-step procedure on how to safely and properly fracture a BHV and report a case of a TAVR in a degenerated rapid deployment valve.

## 1. Introduction

Degeneration of bioprosthetic heart valves (BHVs) requiring a new implant is a featured topic since the use of BHVs is becoming increasingly frequent, overcoming the number of mechanical ones [1–3]. Considering that many patients with degenerated BHVs are at high risk for redo open cardiac surgery, the need for a less-invasive intervention has become a reality and the valve-in-valve (ViV) transcatheter aortic valve replacement (TAVR) has emerged as an effective alternative to redo aortic valve replacement. However, the presence of a smaller-sized surgical BHV may preclude a successful ViV procedure, unless combined approaches, such as balloon valve fracture (BVF), are performed. How to perform a BVF, which are the recommended balloon sizes and balloon pressures required to fracture the frame, and when is the best moment to perform it, if before or after ViV TAVR, are some of the current questions related to this issue. This article aims to provide an updated review of BVF and report an unusual case of TAVR in a previous degenerated rapid deployment prosthesis using the balloon cracking technique.

## 2. Structural Valve Deterioration

The concept of structural valve deterioration (SVD) resulting in severe BHV failure was well defined in the recent published VARC-3 consensus. According to this document, severe hemodynamic valve deterioration means an “increase in mean transvalvular gradient ≥20 mmHg resulting in mean gradient ≥30 mmHg with concomitant decrease in effective orifice area (EOA) ≥0.6 cm^2^ or ≥50% and/or decrease in Doppler velocity index ≥0.2 or ≥40% compared with echocardiographic assessment performed 1–3 months post-procedure, or an increase, or new occurrence, of ≥2 grades, of intraprosthetic aortic regurgitation causing in severe aortic regurgitation” [4].

The options to manage severe SVD are optimized medical therapy (for patients with a low life expectancy for whom any intervention is deemed futile), redo open cardiac surgery, or a transcatheter intervention (ViV TAVR). In the case of the latter, one of the first requirements is to evaluate if the BHV effective orifice has enough size to accommodate a new bioprosthesis implant, or if it is too narrow, which could cause prosthesis-patient mismatch (PPM) [5]. Concerns about final effective orifice area are especially relevant since previous studies have demonstrated that ViV TAVR in patients with small surgical bioprostheses or with pre-existing PPM can result in high residual transvalvular gradients and, consequently, poor clinical outcomes and reduced 1-year survival [6–9]. According to Pibarot et al., PPM occurs when the indexed EOA is <0.85 cm^2^/m^2^ and can be classified as moderate (indexed EOA 0.66–0.85 cm2/m2) or severe (indexed EOA <0.65 cm^2^/m^2^) [10].

Aiming to avoid PPM following ViV TAVR, several strategies have been developed. One possible alternative is to use a transcatheter heart valve (THV) with supra-annular leaflets (e.g., CoreValve Evolut; Medtronic, Minneapolis, MN, USA), which may result in a larger EOA. Another is to deploy the THV in a higher implant depth to improve inflow dynamics and increase the EOA. Additionally, in the presence of a smaller-sized BHV, an effective option is to fracture the previous BHV frame by using a high-pressure balloon [5].

## 3. Preprocedural Planning

Planning a ViV TAVR involves 3 important steps [11]:

1. Careful preprocedural examination of the existing BHV
2. Choice of the new transcatheter heart valve (THV) size and type to be used
3. Assessment if balloon valve fracture is indicated and how to do it

**Figure 1:**
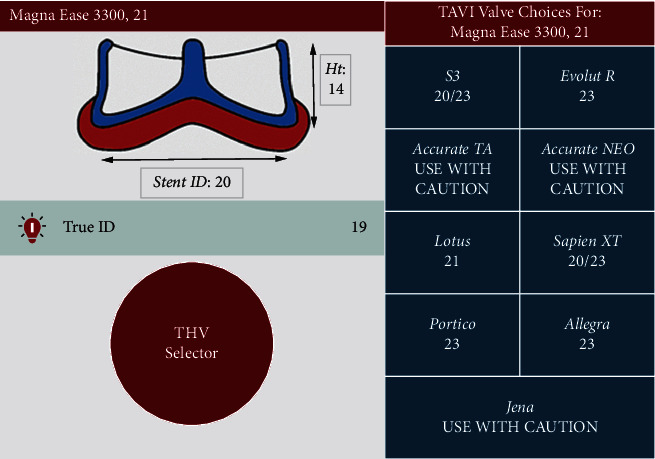
Example of some information obtained in the ViV Aortic App. In this simulation, the true ID for a Magna Ease 21 mm bioprosthesis is 19 mm, and a TAVR using an Evolut R 23 mm self-expanding or a Sapien 3 20/23 mm balloon-expandable would be recommended.

In this line, the size of the degenerate BHV, its model, and true inner diameter (ID) should be checked by analyzing the previous surgical description and the preoperative computed tomography (CT) and confirmed by the intraprocedural fluoroscopic images. With this information, THV selection for ViV TAVR is guided by the true ID of the BHV rather than the labeled surgical valve size. The true ID can be obtained from the manufacturer or from the “ViV Aortic” phone application developed by UBQO Ltd. and Dr. Vinayak Bapat (Figure 1). It is known that for all porcine valves, the true ID is 2 mm smaller than the listed size (i.e., the stented ID), while for pericardial valves, the true ID is 1 mm smaller than the stented ID if the leaflets are mounted inside the stent and equal to the stented ID if the leaflets are mounted outside the stent [12].

## 4. Balloon Valve Fracture

**Figure 2:**
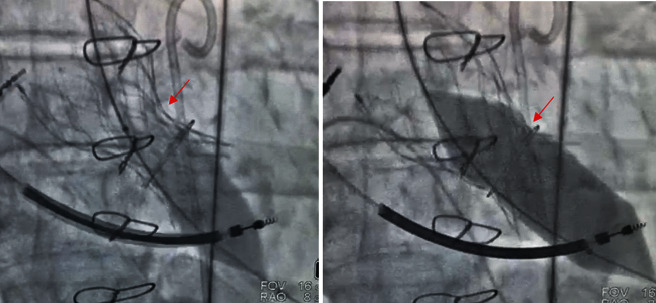
Example of a balloon valve frame fracture using a noncompliant balloon and high-pressure inflation. Observe the prosthesis waist before (first image) and after (second image) the balloon inflation.

BVF is a technique that utilizes high-pressure and noncompliant balloon inflation to fracture a previously implanted surgical valve sewing ring, thus allowing further expansion of the BHV and increasing the maximum EOA (Figure 2) [13]. Therefore, by performing a valve fracture, the inner valve ring of a small BHV could be opened up by a single fracture line enabling a subsequent properly sized THV implantation [14].

In 2017, Allen et al., in a bench testing study, demonstrated that the frame of most, but not all, BHVs can be fractured using high-pressure balloons. According to their tests, Mitroflow, Magna, Magna Ease, Mosaic, and Biocor Epic surgical valves could be successfully fractured using a high-pressure balloon 1 mm larger than the labeled valve size, whereas Trifecta and Hancock II could not be fractured [15]. In this same line, Chhatriwalla and Sorajja showed that some BHVs can be fractured (Biocor Epic, Magna Ease, Mosaic, Mitroflow, Perimount newer generation), others can be significantly remodeled (Inspiris, Carpentier-Edwards Standard, Carpentier-Edwards supra-annular, Perimount old generation, Trifecta), but some prostheses cannot be fractured or remodeled (Avalus and Hancock II) [16].

## 5. Balloon Type, Size, and Pressure

The most frequently used balloons are the noncompliant True Dilatation and Atlas Gold (Bard Peripheral Vascular, Inc., Tempe, AZ, USA).

Traditionally, the balloon is sized 1 mm larger than the true ID; however, as published by Allen et al., successful BVF has been consistently achieved using balloons 1 mm larger than the labeled valve size, which, for most surgical valves, equates to a balloon 3–4 mm larger than the true ID (i.e., a 21 mm valve is fractured with a 22 mm balloon) [13]. These same authors described that balloon pressure required to fracture a stent frame varies from 8 to 24 atmospheres (atm) depending on the type of BHV (Table 1) [15].

Similarly, Johansen et al. have studied an in vitro model, which BHV can be fractured by a high-pressure balloon and what is the pressure required to induce the fracture. The authors observed that valves with a polymer frame were fractured at lower pressures (8–10 atm) than those with a metal stent (19–26 atm). Fracture pressures for the Mosaic valves (19 mm and 21 mm) and the Mitroflow 21 mm valve were in a similar range (8–10 atm). On the other hand, it was not possible to fracture the Trifecta 19 mm, even though the metal frame experienced notable. The Trifecta 21 mm did fracture but at a high pressure of 26 atm. The Magna Ease 19 mm and 21 mm valves, both having metal frames, were considered fracturable [5].

**Figure 3:**
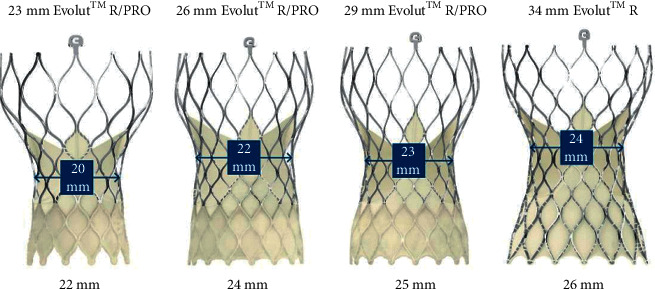
Maximum recommended balloon size for self-expanding CoreValve/Evolut valves (adapted from Chhatriwalla [18]).

More recently, Allen et al. caught the attention to the fact that it is crucial to have an understanding of THV anatomy, particularly when performing BVF after implanting a self-expanding THV. Since the Medtronic self-expanding valve has a narrowed area where the commissures are attached to the nitinol frame, known as the “constrained area,” when using a high-pressure balloon larger than the diameter of the constrained area, operators should be careful to avoid THV trauma, which could lead to severe insufficiency. Thus, according to these authors, when doing BVF with CoreValve/Evolut, a balloon that is no larger than 2 mm of the constrained area is recommended (the waist is 20, 22, 23, and 24 mm, respectively, for CoreValve Evolut Pro/R 23, 26, 29, and 34 mm THV). Furthermore, ideally, the proximal shoulder of the balloon should be placed distal to the CoreValve (Figure 3). Exemplifying, the 21 mm Magna valve should be fractured with 22 mm balloon if a 23 mm CoreValve is used [15]. On the other hand, when using a balloon-expandable valve, the goal is to size the balloon considering the perfect THV expansion; therefore, a 23 mm Sapien valve should be fractured using a 23 mm balloon [17]. Left ventricular outflow tract, coronary sinuses, and sinotubular junction sizes and calcification should also be carefully assessed when evaluating BVF suitability.

As commented above, initial *in vitro* testing has demonstrated that BVF results in an increase of 3–4 mm in the ID of surgical valves with labeled valve sizes of 19 and 21 mm, respectively. Moreover, according to a recent publication from Allen et al., additional bench testing has shown that an expansion of 5 mm can be achieved in larger labeled valve sizes (23 and 25 mm), and clinical experience suggests that even a 6 mm increase in diameter can be obtained following BVF in larger (≥27 mm) surgical valves [17].

## 6. Inflation Technique

The setup for high-pressure balloon inflation includes the following:

- Noncompliant balloon
- 60 mL Luer-Lok syringe filled with dilute contrast
- Inflation device
- High-pressure stopcock

The technique consists of placing the noncompliant balloon within the surgical prosthesis, and then, during rapid ventricular pacing, the balloon is inflated by hand using the 60 mL syringe with diluted contrast. The stopcock is opened to the inflation device, and the balloon pressure is increased to the fracture threshold. BVF is noted by a sudden drop in the inflation pressure on the inflation device gauge and a visible release of the balloon waist, which is frequently accompanied by an audible “click,” visual and haptic feedback. Successful BVF is noted fluoroscopically as a release of the balloon waist, but this is not always obvious. The valve is then echocardiographically assessed, and repeat hemodynamic measurements are obtained to ensure optimal expansion and satisfactory drop in transvalvular gradients. If the mean gradient is still elevated and the valve was not fractured, the maneuver can be carefully repeated. If gradients remain elevated after successful BVF, postdilation may be performed by inflation of a slightly larger balloon [19].

Taking into consideration that prolonged rapid pacing is required during BVF, it may be advisable to perform the procedure under general anesthesia. In addition, general anesthesia provides a more controlled environment during the procedure and eventful complications management. Transesophageal echocardiography guidance has been also recommended since it can be used to evaluate adequate THV expansion and leaflet excursion and detect potential complications early [20].

## 7. Time to Perform Balloon Fracture

BVF can be performed before or after THV deployment. The choice involves a balance between the potential risk of inducing a catastrophic valve insufficiency versus the unknown influence of high-pressure balloon inflation on the THV leaflets' structural integrity and, consequently, its long-term durability. Besides, BVF before may be effective to fracture the surgical valve but not to ensure adequate THV expansion. This is particularly true with balloon-expandable valves, whose compliant delivery balloon does not generate sufficient pressure to fully expand the THV in a fractured surgical valve [15]. In this same line, in vivo tests showed that degenerated surgical valves may impede even self-expanding THVs from fully expanding when using the BVF-first strategy. Therefore, to maximize the increase in diameter achieved with BVF, the THV itself needs to be dilated with high-pressure balloon inflation (as occurs with BVF after TAVR) [13]. A crucial point when performing BVF before is to assure that the THV is ready for prompt use if acute regurgitation occurs.

In 2019, Allen et al. evaluated the results of 75 patients who underwent BVF at 21 centers. BVF was performed successfully in 100% of them, with an in-hospital and/or 30-day mortality of 2.7% (2 out of 75) and no case of annular/aortic root rupture, coronary occlusion, or new pacemaker implant. The final mean transvalvular gradient was 9.2 ± 6.3 mmHg and was significantly lower when BVF was performed after compared with BFV before TAVR (8.1 ± 4.8 mmHg vs 16.9 ± 10.1 mmHg; *p* < 0.001). In a hierarchical multiple linear regression analysis, performing BVF after ViV TAVR (*p* < 0.001) and performing BVF with a balloon that was at least 3 mm larger than the true ID of the surgical valve (*p*=0.038) were the only procedural factors associated with a lower final mean gradient. Therefore, the authors concluded that BVF performed after ViV TAVR and using larger balloons contributed to achieving the best hemodynamic results [13]. Following this study, many centers have adopted the technique of performing BVF after ViV TAVR.

## 8. THV Selection

Selection of THV size is not always straightforward when BVF is performed because the size of the THV should be based on the anticipated increase in the true ID of the surgical valve. The question remains whether to use a THV that can be optimally expanded after BVF or to upsize to a larger THV, anticipating achieving a larger EOA and superior hemodynamic results. Bench testing has suggested that a larger prosthesis, even if expanded to a less than nominal diameter, may result in a more favorable transvalvular gradient. On the other hand, Allen et al. have shown that upsizing the THV did not result in a difference in the final mean gradient or EOA after BVF. These findings suggest that if there is any hemodynamic downside to using intra-annular THVs during ViV TAVR, it may be overcome by performing BVF and optimally expanding the THV [13].

Another uncertainty point is the decision between self-expanding or balloon-expandable THV, with some data suggesting that self-expanding THVs could result in superior procedural hemodynamics and increased EOA compared with the balloon-expandable one [21, 22].

## 9. Indications

The indications to perform the BVF technique are not fully defined. The majority of patients, in particular those with large surgical valves, are likely to achieve adequate hemodynamic results after a standard ViV TAVR, and patients without PPM have an excellent 1-year survival. Therefore, patients who stand to benefit the most from BVF are those who are predisposed to PPM and high residual transvalvular gradients, including those with small BHVs (labeled valve size ≤21 mm) and/or stenosis as the BHV failure mechanism. Whether patients with large BHVs (>21 mm labeled valve size) or intermediate transvalvular gradients (10–20 mmHg) stand to benefit from BVF is still not known [20]. Besides this classical BVF indication (increase final valve diameter and decrease residual transvalvular gradient), the procedure has been also considered to optimize THV expansion, manage perivalvular leak (PVL), prevent the constrained THV from pinwheeling, and potentially improve THV durability.

## 10. Concerns

It is important to acknowledge that the clinical experience with BVF is still early [20] and there are some theoretical risks associated with BVF such as acute severe aortic regurgitation causing hemodynamic collapse, THV migration, coronary obstruction, aortic root injury, and THV failure due to balloon injury to the leaflets [23].

Saxon et al. highlighted that with BVF the architecture of the BHV is altered such that the final position of the BHV leaflets is less certain. These authors also commented that the additional space in the coronary sinuses necessary to accommodate BVF is not fully understood. Extrapolating from the recommended safety margins of ViV TAVR, it is reasonable to estimate that a BHV to coronary distance of less than 5 mm could be considered to place a patient at high risk for coronary occlusion when BVF is performed [20].

Furthermore, it should be highlighted that BVF does not completely extinguish the risk of PPM. While BVF has been shown to enlarge the neoannulus by approximately 3 mm, “shoehorning” a larger THV into the annulus may even distort the valve [13].

## 11. Case Report—TAVR in a Rapid Deployment Valve

Although TAVR is a well-established treatment option for severe symptomatic native aortic valve stenosis, BHV failure [24], and even for TAVR failure [25], there is almost no data supporting TAVR in degenerated rapid deployment valves. Here, we describe a case of rapid deployment valve failure that was treated with TAVR and balloon cracking.

**Figure 4:**
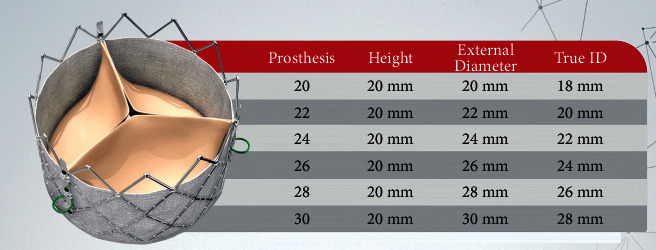
Chart of Inovare® Alpha sizes.

A 79-year-old female patient with 85 kg (body surface are = 2 cm^2^) had a rapid deployment aortic valve (Inovare® Alpha 22 mm; Braile Biomédica, Brazil) implanted 7 years ago (Figure 4). Three years after the first surgery, she was submitted to a percutaneous balloon dilatation aiming to treat a moderate aortic regurgitation (AR) due to PVL. The mean aortic valve gradient at the index procedure was around 20 mmHg.

Currently, she presented with heart failure due to nonstructural and structural valve degeneration (severe PVL, severe central aortic regurgitation, and severe stenosis with a mean gradient of 46 mmHg). Angio CT showed a true ID of 18 mm and thickened leaflets. Left coronary artery height was 8 mm, and the VTC was 5 mm. Femoral accesses are judged adequate.

**Figure 5:**
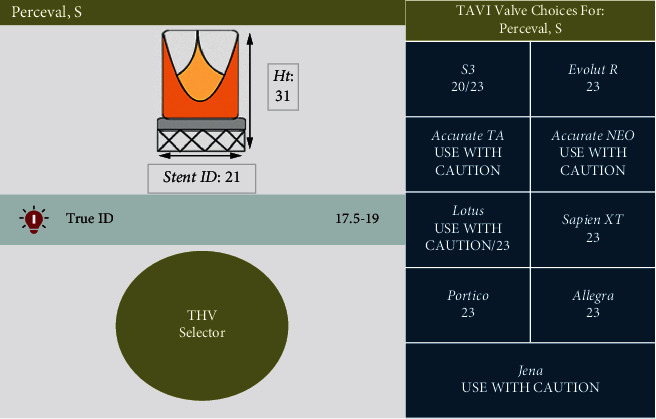
As Inovare® Alpha is not present in the ViV App, we looked at the Perceval valve, which has a similar true ID. In a true ID of 17.5–19 mm, an Evolut R 23 mm is suggested.

**Figure 6:**
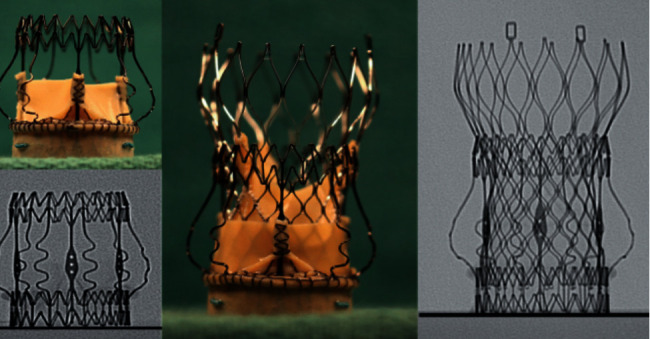
ViV App suggestion of depth of implantation of an Evolut R 23 mm in a Perceval valve.

**Figure 7:**
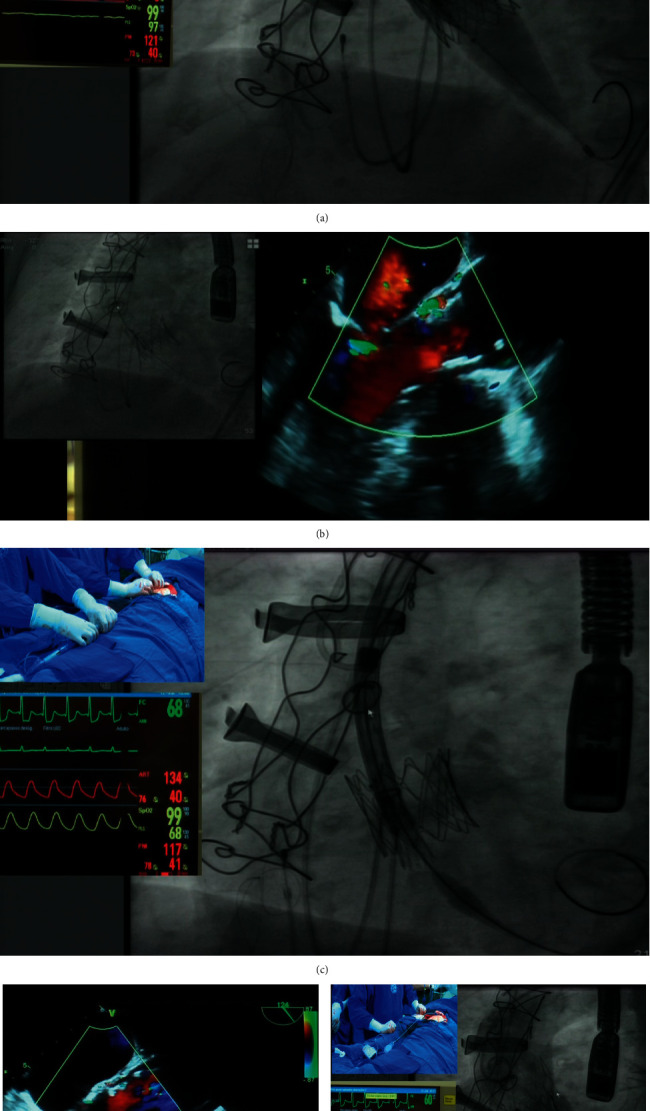
Step-by-step procedure. (a) Predilatation and valve cracking using an Atlas 20 mm noncompliant balloon while injecting contrast in the ascending aorta to simultaneously evaluate the left coronary artery flow. (b) Immediate prosthesis cracking and expansion with mean gradient reduction and PVL resolution. (c) Slow Evolut R 23 deployment immediately below the previously implanted rapid deployment valve. (d) Aortic regurgitation reduction on TEE and expansion of the previously implanted rapid deployment valve. (e) Final aortogram showing no aortic regurgitation and both prostheses proper expansion with a mean gradient of 8 mmHg.

The patient was considered at high surgical risk for a redo surgery, and thus a TAVR was indicated. The procedure was performed throughout percutaneous transfemoral access, and an Evolut R 23 mm THV (Medtronic, USA) was deployed using the balloon cracking technique to optimize the THV expansion and reduce final gradients (Figures 5–7).

## 12. Step-by-Step Procedure

1. The procedure was carried out under general anesthesia and transesophageal echocardiogram (TEE) guidance
2. A Lunderquist double curve guidewire was placed in the left ventricle
3. We decided to predilate the previous rapid deployment Inovare® Alpha prosthesis and crack it with a noncompliant 20 mm Atlas balloon with simultaneous injection of contrast in the ascending aorta. After this maneuver, echocardiogram and invasive measurements showed an excellent result, with elimination of the aortic regurgitation.
4. An Evolut R 23 mm was implanted, and a mean gradient of 8 mmHg was measured at the end of the procedure.

Implantation of a TAVR within a rapid deployment prosthesis is a new procedure and poses several challenges. This patient had multifactorial problems such as small prosthesis with some degree of PPM, valve regurgitation and stenosis (structural and nonstructural), and PVL. Predilatation using a noncompliant Atlas balloon was crucial to reduce the aortic regurgitation, and fracture the previous rapid deployment valve resulted in a significant final mean gradient reduction. As mentioned by Tarantini et al., sutureless and stentless surgical aortic valves cannot undergo BVF; however, sutureless valves can potentially be remodeled by overexpansion [23].

## 13. Conclusion

BVF of a previously implanted stented bioprosthetic valve is an important tool to reduce the aortic valve gradient and the risk of PPM. We presented a case in which a TAVR was deployed in a small and degenerated rapid deployment prosthesis using the balloon cracking technique. The employment of the balloon cracking technique in the setting was really useful to reduce aortic regurgitation and final gradients. Further studies are necessary to confirm this anecdotal initial result.

## Figures and Tables

**Table 1:** Balloon fracture pressures according to Allen et al., 2017 [15]. In this study, the balloon was sized 1 mm larger than the valve size. atm, atmospheres.

Valve type	TRUE balloon	Atlas Gold balloon
	Fracture pressure	Fracture pressure
St. Jude Trifecta		
19 mm	No	No
21 mm	No	No

St. Jude Biocor Epic		
21 mm	8 atm	8 atm

Medtronic Mosaic		
19 mm	10 atm	10 atm
21 mm	10 atm	10 atm

Medtronic Hancock II		
21 mm	No	No

Sorin Mitroflow		
19 mm	12 atm	12 atm
21 mm	12 atm	12 atm

Edwards Magna		
19 mm	24 atm	24 atm
21 mm	24 atm	24 atm

Edwards Magna Ease		
19 mm	18 atm	18 atm
21 mm	18 atm	18 atm

## References

[1] Ciubotaru A., Cebotari S., Tudorache I., Beckmann E., Hilfiker A., Haverich A. (2013). Biological heart valves. *Biomedizinische Technik/Biomedical Engineering*.

[2] Joint Task Force on the Management of Valvular Heart Disease of the European Society of Cardiology (ESC), European Association for Cardio-Thoracic Surgery (EACTS), Vahanian A. (2012). Guidelines on the management of valvular heart disease (version 2012). *European Heart Journal*.

[3] Nishimura R. A., Otto C. M., Bonow R. O. (2014). 2014 AHA/ACC guideline for the management of patients with valvular heart disease: a report of the American college of cardiology/American heart association task force on practice guidelines. *Circulation*.

[4] VARC-3 Writing Committee, Généreux P., Alu M. C. (2021). Valve academic research consortium 3: updated endpoint definitions for aortic valve clinical research. *Journal of the American College of Cardiology*.

[5] Johansen P., Engholt H., Tang M., Nybo R., Rasmussen P., Nielsen-Kudsk J. E. (2017). Fracturing mechanics before valve-in-valve therapy of small aortic bioprosthetic heart valves. *EuroIntervention*.

[6] Dvir D., Webb J., Brecker S. (2012). Transcatheter aortic valve replacement for degenerative bioprosthetic surgical valves: results from the global valve-in-valve registry. *Circulation*.

[7] Dvir D., Webb J. G., Bleiziffer S. (2014). Transcatheter aortic valve implantation in failed bioprosthetic surgical valves. *JAMA*.

[8] Faerber G., Schleger S., Diab M. (2014). Valve-invalve transcatheter aortic valve implantation: the new playground for prosthesispatient mismatch. *Journal of Interventional Cardiology*.

[9] Pibarot P., Simonato M., Barbanti M. (2018). Impact of pre-existing prosthesis-patient-mismatch on survival following aortic valve-in-valve procedures. *JACC: Cardiovascular Interventions*.

[10] Pibarot P., Magne J., Leipsic J. (2019). Imaging for predicting and assessing prosthesis-patient mismatch after aortic valve replacement. *JACC Cardiovasc Imaging*.

[11] Perri J. L., Blusztein D., Mahadevan V. S., Nguyen T. C. (2021). Fracture of a 21 mm failed bioprosthetic aortic valve. *Annals of Cardiothoracic Surgery*.

[12] Bapat V. N., Attia R., Thomas M. (2014). Effect of valve design on the stent internal diameter of a bioprosthetic valve. *JACC: Cardiovascular Interventions*.

[13] Allen K. B., Chhatriwalla A. K., Saxon J. T. (2019). Bioprosthetic valve fracture: technical insights from a multicenter study. *Journal of Thoracic and Cardiovascular Surgery*.

[14] Nielsen-Kudsk J. E., Christiansen E. H., Terkelsen C. J. (2015). Fracturing the ring of small mitroflow bioprostheses by high-pressure balloon predilatation in transcatheter aortic valve-in-valve implantation. *Circulation: Cardiovascular Interventions*.

[15] Allen K. B., Chhatriwalla A. K., Cohen D. J. (2017). Bioprosthetic valve fracture to facilitate transcatheter valve-in-valve implantation. *Annals of Thoracic Surgery*.

[16] Chhatriwalla A. K., Sorajja P. (2018). Expanding indications for bioprosthetic valve fracture and bioprosthetic valve remodeling. *Circulation: Cardiovascular Interventions*.

[17] Allen K. B., Chhatriwalla A. K., Saxon J. T. (2021). Bioprosthetic valve fracture: a practical guide. *Annals of Cardiothoracic Surgery*.

[18] Chhatriwalla A. K. (2019). *Fracturing and Remodeling Bioprosthetic Valves*.

[19] Almomani A., Chhatriwalla A. K. (2019). Bioprosthetic valve fracture during ViV TAVR. A step-by-step practical guide for performing BVF to facilitate ViV TAVR. *Cardiac Interventions Today*.

[20] Saxon J. T., Allen K. B., Cohen D. J., Chhatriwalla A. K. (2017). Bioprosthetic valve fracture during valve-in-valve TAVR: bench to bedside. *Interventional Cardiology Review*.

[21] Simonato M., Azadani A. N., Webb J. (2016). In vitro evaluation of implantation depth in valve-in-valve using different transcatheter heart valves. *EuroIntervention*.

[22] Azadani A. N., Reardon M., Simonato M. (2017). Effect of transcatheter aortic valve size and position on valve-invalve hemodynamics: an in vitro study. *Journal of Thoracic and Cardiovascular Surgery*.

[23] Tarantini G., Dvir D., Tang G. H. L. (2021). Transcatheter aortic valve implantation in degenerated surgical aortic valves. *EuroIntervention*.

[24] Nalluri N., Atti V., Munir A. B. (2018). Valve in valve transcatheter aortic valve implantation (ViV-TAVI) versus redo-surgical aortic valve replacement (redo-SAVR): a systematic review and meta-analysis. *Journal of Interventional Cardiology*.

[25] Barbanti M., Webb J. G., Tamburino C. (2016). Outcomes of redo transcatheter aortic valve replacement for the treatment of postprocedural and late occurrence of paravalvular regurgitation and transcatheter valve failure. *Circulation: Cardiovascular Interventions*.

